# Development and Characterization of Hollow-Shell Collagen Microcapsules for Three-Dimensional Cell Culture

**DOI:** 10.3390/gels12010015

**Published:** 2025-12-24

**Authors:** Yusuke Chiwata, Shigehisa Aoki, Takehisa Sakumoto, Takayuki Narita

**Affiliations:** 1 Department of Chemistry and Applied Chemistry, Faculty of Science and Engineering, Saga University, Saga 840-8502, Japan; 2 Department of Pathology and Microbiology, Faculty of Medicine, Saga University, Saga 840-8502, Japan

**Keywords:** collagen microcapsules, hollow-shell structure, 3D cell culture, cell encapsulation, NIH 3T3 fibroblasts, tissue engineering, hydrogel scaffolds

## Abstract

Three-dimensional (3D) cell culture systems require biocompatible carriers that provide both structural support and efficient mass transport. Conventional alginate-based encapsulation systems suffer from poor molecular diffusion, lack of cell adhesion motifs, and structural instability under physiological conditions. Here, we report the first development of hollow-shell collagen microcapsules (CMCs) fabricated via a gelatin sacrificial template approach that overcomes these critical limitations. The hollow architecture combined with collagen’s intrinsic bioactivity achieved 2.5-fold enhancement in molecular permeability compared to conventional alginate beads, while maintaining structural integrity for 14 days versus 3-day collapse of alginate controls. NIH 3T3 fibroblasts encapsulated within CMCs demonstrated superior proliferation and formed tissue-like multilayered structures with extensive extracellular matrix deposition. This platform represents a significant advance in 3D cell culture technology, providing a biologically functional microenvironment with enhanced mass transport properties for applications in tissue engineering and regenerative medicine.

## 1. Introduction

Three-dimensional (3D) cell culture technologies have emerged as essential tools to overcome the limitations of traditional two-dimensional (2D) monolayer culture, which fails to recapitulate the complex architecture, biochemical gradients, and cell–cell/matrix interactions present in native tissues [1]. Among these technologies, microencapsulation of living cells within semi-permeable hydrogel matrices has attracted increasing attention for applications in tissue engineering [2], regenerative medicine [3], cell-based therapy [4], drug screening [5], and disease modeling [6]. Encapsulated systems provide a protective environment [5], allow controlled mass transport of nutrients and metabolites, and can potentially mimic in vivo-like microenvironments [7].

Hydrogels based on alginate have long been employed as cell-encapsulating carriers because of their biocompatibility, low cost, and ability to gel under mild ionic crosslinking conditions [8,9]. Nevertheless, typical alginate beads possess a fully gel-filled interior, which limits the diffusion of oxygen and metabolites, thereby imposing transport barriers that hinder long-term cell survival [10]. Furthermore, alginate inherently lacks cell adhesion motifs, leading to poor cell anchorage and altered functionality [11]. Even when modified with adhesion peptides or blended with extracellular matrix (ECM) proteins, alginate matrices remain structurally unstable under physiological conditions; calcium ions required for crosslinking are gradually chelated or exchanged in culture medium, frequently resulting in bead swelling, deformation, or rupture [12,13]. These drawbacks highlight the need for alternatives that more closely emulate the biochemical and biomechanical characteristics of native ECM.

Collagen, the most abundant structural protein in mammalian tissues, has attracted attention as a naturally derived biomaterial capable of supporting cell attachment, proliferation, and matrix remodeling [14,15,16]. Type I collagen is particularly advantageous because it undergoes fibrillogenesis at neutral pH and physiological temperature without the requirement for photoinitiators or cytotoxic chemicals, and because it contains integrin-binding domains that facilitate cell adhesion [16,17]. However, despite these intrinsic biological merits, the direct application of collagen to cell encapsulation has been hindered by several unresolved technical challenges. Collagen solutions are typically supplied in strongly acidic form (pH < 3), and direct neutralization in the presence of cells can induce local pH fluctuations and osmotic stress, severely compromising cell viability [18]. Furthermore, collagen hydrogels exhibit low mechanical strength and are highly hydrated, [14] making them susceptible to deformation and rupture during handling or culture, especially when processed into microscale capsules [19]. Conventional collagen microgels usually adopt a core-filled configuration; the dense fibrillar network throughout the interior restricts molecular diffusion and leaves insufficient free space for cellular proliferation or tissue-like organization [20,21]. Attempts to form hollow collagen capsules often result in non-uniform shells, incomplete removal of the core material, or structural collapse due to weak interfacial stability. In addition, post-gelation loading of cells leads to shell disruption or cell leakage [22], whereas pre-gel encapsulation exposes cells to acidic or enzymatic environments during pH adjustment, resulting in cytotoxicity [18]. These issues have collectively impeded the development of collagen-based microcapsules that are both biologically functional and structurally robust.

To address these limitations, we developed a novel fabrication strategy for hollow collagen microcapsules (CMCs) based on a thermally responsive gelatin sacrificial template. This approach enables cell encapsulation under mild conditions: gelatin droplets containing living cells are first formed, coated with neutralized type I collagen, and then incubated at 37 °C. During incubation, the collagen shell undergoes thermal gelation while the gelatin core simultaneously liquefies (gel-to-sol transition) and diffuses outward through the permeable collagen membrane, yielding a cell-laden hollow structure without exposing cells to acidic environments or cytotoxic crosslinkers. This gelatin sacrificial template mechanism enables hollow cavity formation without compromising membrane permeability; indeed, the collagen shell exhibits high molecular diffusivity as demonstrated in subsequent permeability measurements. This method is expected to combine the biochemical functionality of collagen with the structural advantages of a hollow architecture, potentially enabling improved nutrient diffusion, enhanced cell adhesion, and sufficient intraluminal space for cell growth and ECM production.

The aim of this study is to systematically evaluate the physicochemical and biological properties of these CMCs and compare them with conventional core-filled alginate beads. Particular emphasis is placed on membrane thickness uniformity, molecular permeability, cell proliferation dynamics, long-term structural stability, and histological tissue organization within the hollow lumen. We demonstrate that the proposed CMC system overcomes major limitations of existing hydrogel-based carriers and provides a promising platform for next-generation 3D cell culture, with potential applications in regenerative medicine, organoid engineering, and drug testing.

## 2. Results and Discussion

### 2.1. Structural Characterization of CMCs and Alg-Beads

**Figure 1:**
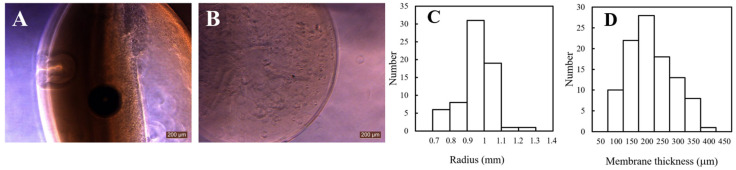
Structural characterization of collagen microcapsules (CMCs) and alginate beads (Alg-Beads). (**A**) Phase-contrast microscopic image of a CMC showing distinct hollow-shell architecture with a cell-laden interior. Scale bar: 200 µm. (**B**) Phase-contrast microscopic image of an alginate bead showing core-filled structure. Scale bar: 200 µm. (**C**) Radius distribution of CMCs. (**D**) Membrane thickness distribution of CMCs.

Figure 1 shows representative phase-contrast microscopic images of freshly prepared collagen microcapsules (CMCs) and alginate beads (Alg-Beads), along with quantitative analysis of the CMC membrane thickness distribution. As seen in Figure 1A, CMCs exhibited a distinct hollow-shell architecture with a visible interior lumen and a well-defined, translucent collagen membrane surrounding the void space. The cells were initially dispersed throughout the gelatin-collagen mixture during fabrication and subsequently became localized near the inner surface of the collagen shell after the gelatin core liquefied and diffused outward. This structural configuration is fundamentally different from conventional core-filled hydrogel carriers.

In contrast, Alg-Beads (Figure 1B) displayed a fully filled internal structure, where cells were uniformly distributed throughout the dense alginate matrix. This core-filled morphology is characteristic of conventional hydrogel-based encapsulation systems and has been widely reported in the literature [23,24]. However, the lack of an internal void space in Alg-Beads imposes significant diffusion limitations, as nutrients, oxygen, and metabolic waste must traverse the entire gel matrix to reach or exit from cells located deep within the bead interior [10,21].

To quantitatively evaluate the structural uniformity of the CMCs, the membrane thickness and capsule size were measured at multiple positions immediately after fabrication (Day 0). Figure 1C presents a quantitative analysis of the capsule radius distribution for CMCs, showing a mean radius of 0.95 ± 0.1 mm (corresponding to a mean diameter of 1.9 ± 0.2 mm), indicating excellent reproducibility in size control during the droplet formation process. Figure 1D shows the membrane thickness distribution of CMCs, with a mean thickness of 173 ± 37 µm and values ranging predominantly from 100 to 250 µm. Representative images of multiple CMCs demonstrating fabrication reproducibility are shown in Supplementary Figure S1. The relatively narrow distribution indicates good reproducibility and structural consistency of the fabrication process. The observed thickness is within an optimal range for efficient molecular diffusion while maintaining adequate mechanical integrity [21,25,26]. Statistical analysis revealed no significant correlation between capsule radius and membrane thickness, indicating that membrane formation is independent of overall capsule size within the examined range. This size-independent membrane formation is advantageous for reproducibility and scalability of the fabrication process. The alginate beads (Alg-Beads) used as controls exhibited a mean diameter of 1.6 ± 0.1 mm, consistent with our previous report [27].

The presence of a well-defined and relatively thin membrane is of particular significance. First, it minimizes the diffusion path length for nutrient and oxygen supply, thereby reducing the risk of nutrient depletion and hypoxic conditions that commonly occur in thick hydrogel constructs. Second, the hollow lumen provides ample free space for cellular proliferation, migration, and extracellular matrix deposition, which are essential for tissue-like organization and long-term viability. Third, the uniform thickness ensures consistent permeability and mechanical properties across different capsules, which is critical for reproducibility in experimental and clinical applications.

### 2.2. Diffusion of Fluorescent Probes

**Figure 2:**
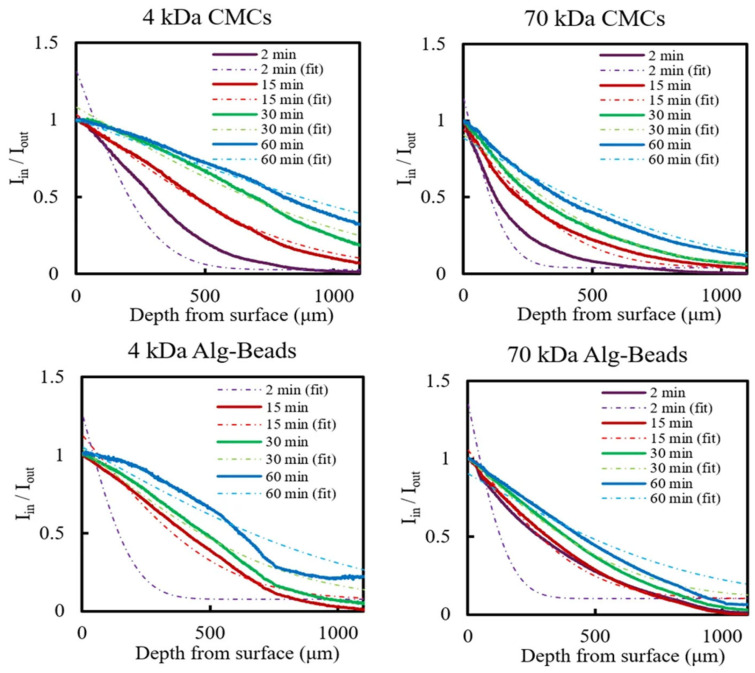
Representative normalized fluorescence intensity profiles (*I*_in_/*I*_out_) and fitted diffusion curves for FITC-dextran (4 kDa and 70 kDa) in CMCs and Alg-Beads at multiple time points.

To further elucidate how the hollow-shell architecture influences mass transport, we evaluated the permeability of CMCs and Alg-Beads by monitoring the inward diffusion of FITC-labeled dextran molecules with two distinct molecular weights (4 kDa and 70 kDa). These molecular weights were selected to represent small metabolites and peptides and larger molecular weight solutes, respectively, providing size-based models for evaluating the permeability characteristics relevant to cell culture and therapeutic applications. Representative normalized fluorescence intensity profiles and the corresponding diffusion coefficients are presented in Figure 2 and Table 1.

For 4 kDa FITC–dextran, CMCs exhibited a diffusion coefficient of approximately 219.2 ± 2.9 µm^2^/s, which was significantly higher than that of Alg-Beads (98.8 ± 2.3 µm^2^/s). This nearly 2.1-fold enhancement in diffusion rate can be attributed primarily to the absence of a dense gel matrix in the hollow interior of CMCs. In Alg-Beads, the diffusing molecules must traverse the entire thickness of the alginate gel network, where the crosslinked polymer chains impose substantial steric hindrance and frictional drag [28]. In contrast, the hollow lumen of CMCs allows for rapid convective and diffusive transport in the void space, effectively reducing the overall diffusion resistance.

When the molecular weight was increased to 70 kDa, diffusion in both systems was markedly reduced. In CMCs, the diffusion coefficient decreased to 66.4 ± 0.6 µm^2^/s, while in Alg-Beads it dropped to 69.8 ± 2.2 µm^2^/s. Although both carriers exhibited a size-dependent decrease in permeability, CMCs maintained a 2.5-fold higher diffusion coefficient than Alg-Beads for the smaller 4 kDa probe, demonstrating the advantage of the hollow architecture for enhancing small molecule transport. The observed molecular sieving effect indicates that the collagen membrane functions as a selective barrier, permitting rapid passage of small solutes while partially restricting the diffusion of large macromolecules. This property can be advantageous for controlling the release kinetics of therapeutic agents or for retaining secreted factors produced by encapsulated cells [29]. It should be noted that while FITC-dextran provides a well-characterized and photostable probe for permeability measurements based on molecular size, the specific diffusion behavior of globular proteins, antibodies, and other biomolecules may differ due to differences in molecular conformation, charge, and interactions with the matrix. Nevertheless, the size-based approximation provided by dextran molecular weight standards offers valuable quantitative insight into the molecular transport properties and size-selectivity of the capsule membranes under physiologically relevant conditions.

Combined with the membrane thickness distribution shown in Figure 1C, these findings confirm that the hollow-shell architecture of CMCs provides a substantial improvement in mass transfer efficiency compared to conventional core-filled hydrogel carriers. The thin collagen membrane and hollow interior collectively minimize diffusion resistance, thereby ensuring adequate nutrient supply and waste removal, even during prolonged culture periods. This enhanced permeability is expected to support higher cell densities, improved cell viability, and more robust tissue-like organization within the capsules. While our functional permeability measurements provide valuable insights into molecular transport properties, detailed structural characterization of the alginate bead porosity using advanced techniques such as cryo-scanning electron microscopy or small-angle scattering methods would further elucidate the mechanistic basis for the observed differences in diffusion behavior. Such comparative structural analysis represents an important direction for future investigation.

### 2.3. Cell Proliferation Capacity and Structural Stability of Carriers

**Figure 3:**
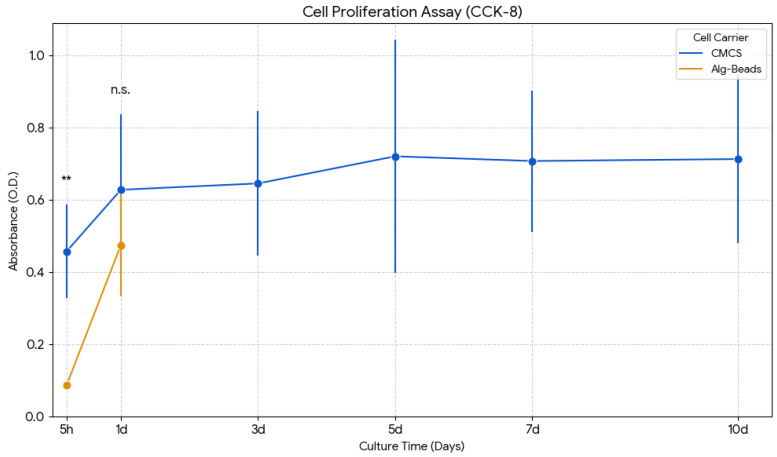
Proliferation of NIH 3T3 fibroblasts within different carriers as assessed by the CCK-8 assay. Absorbance at 450 nm is presented as mean ± SD (*n* = 6) at 5 h and on Days 1, 3, 5, 7, and 10. ** *p* < 0.01 vs. CMCs. Structural collapse of Alg-Beads prevented further measurement beyond Day 1.

To assess the biological performance of the hollow CMCs as a three-dimensional cell culture scaffold, we monitored the proliferation of encapsulated NIH 3T3 fibroblasts over a 10-day culture period using the CCK-8 metabolic activity assay. Parallel experiments were conducted with Alg-Beads to provide a direct comparison with a widely used core-filled carrier system. The temporal evolution of absorbance at 450 nm, which reflects cell metabolic activity and number, is presented in Figure 3.

A pronounced difference was observed at the early attachment stage (5 h). The absorbance of the CMC group was significantly higher than that of the Alg-Beads group, indicating that cells encapsulated in CMCs exhibited more rapid initial metabolic activation. This enhanced early-stage response can be attributed to the presence of collagen, which possesses intrinsic cell adhesion motifs such as RGD sequences and integrin-binding domains [30,31]. These biochemical cues facilitate cell attachment to the collagen matrix and trigger intracellular signaling cascades that promote cell spreading, cytoskeletal reorganization, and activation of proliferation-related pathways [32]. In contrast, alginate lacks these adhesion ligands, resulting in poor initial cell anchorage and lower metabolic activity [33].

By Day 1, the absorbance of Alg-Beads increased sharply to 0.475 ± 0.142, becoming comparable to that of CMCs (0.498 ± 0.089). This temporary convergence suggests that cells in both systems adapted to their respective microenvironments and began to proliferate. However, this apparent parity was short-lived.

Despite this transient increase in cell activity, Alg-Beads exhibited severe structural instability. By Day 3, the alginate matrix began to swell and deform due to the gradual loss of calcium ions, which are essential for maintaining the ionic crosslinks that stabilize the gel network. This phenomenon, known as calcium ion exchange, occurs when Ca^2+^ ions are chelated by phosphate or citrate ions present in culture medium, or are replaced by monovalent cations (Na^+^, K^+^) that do not support alginate gelation. As a result, the crosslinked structure weakens, leading to bead swelling, loss of spherical shape, and eventually rupture [24]. Microscopic observation revealed that the majority of Alg-Beads had collapsed by Day 3, releasing the encapsulated cells into the surrounding medium. Consequently, the CCK-8 absorbance values for Alg-Beads dropped sharply after Day 3, reflecting both the loss of cells from the ruptured beads and the inability to sustain a viable 3D microenvironment.

In contrast, CMCs preserved their spherical shape and shell integrity throughout the 10-day culture period. The absorbance of the CMC group continued to increase progressively from Day 1 to Day 10, reaching a peak value of 0.923 ± 0.176 on Day 10. This sustained increase in metabolic activity indicates robust cell proliferation within the hollow lumen and along the inner surface of the collagen membrane. Importantly, phase-contrast microscopy confirmed that CMCs remained structurally intact without any signs of swelling, rupture, or disintegration, even after prolonged culture. This exceptional stability can be attributed to the covalent crosslinking and fibrillar network formed during collagen fibrillogenesis, which provides superior mechanical strength compared to the ionic crosslinking in alginate gels.

These findings clearly demonstrate two major advantages of CMCs over Alg-Beads: (1) superior initial cell adhesion, due to collagen-mediated integrin interactions; (2) outstanding long-term structural stability, enabling sustained cell proliferation without rupture. Together with the enhanced permeability and optimized membrane thickness described in previous sections, these results establish CMCs as a highly promising platform for long-term 3D cell culture and tissue engineering applications. Future studies comparing cell behavior within CMCs to conventional 2D culture and bulk 3D collagen gels would provide additional context for comprehensively evaluating the advantages of the hollow microcapsule architecture. Such comparative analysis would further establish the utility of the CMC platform across different tissue engineering applications.

### 2.4. Histological Evaluation of CMCs After Long-Term Culture

**Figure 4:**
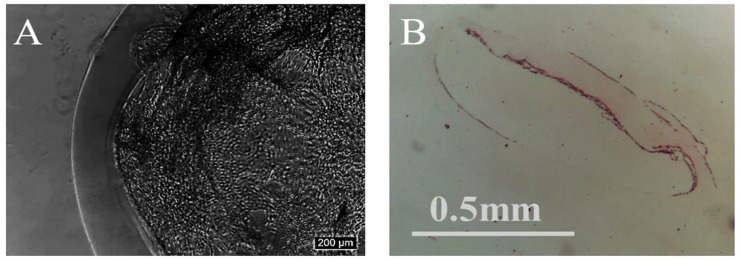
Histological evaluation of CMCs cultured for 14 days. (**A**) Phase-contrast microscopic image showing dense cellular colonization along the inner shell. (**B**) H&E-stained cross-section showing well-defined collagen membrane with viable cells and extracellular matrix deposition. Scale bars: 200 µm (**A**), 0.5 mm (**B**).

To further assess the structural integrity of the CMCs and the cellular organization within the capsules after extended culture, we performed histological analysis on Day 14. CMCs were fixed, sectioned, and stained with hematoxylin and eosin (H&E) to visualize cellular morphology, tissue architecture, and extracellular matrix deposition. Representative images are presented in Figure 4.

#### 2.4.1. Cellular Morphology and Organization in Intact CMCs

Phase-contrast microscopy (Figure 4A) revealed that NIH 3T3 fibroblasts densely colonized the inner surface of the collagen shell, forming a continuous, multilayered cellular lining along the lumen. Cells exhibited elongated, spindle-shaped morphology characteristic of adherent fibroblasts, and displayed extensive cell–cell contacts, suggesting active intercellular communication and coordinated tissue-like organization. Notably, the hollow interior remained largely free of debris or necrotic material, indicating sustained cell viability and efficient removal of metabolic waste throughout the culture period.

#### 2.4.2. Histological Structure and ECM Deposition

Hematoxylin and eosin (H&E) staining of sectioned CMCs (Figure 4B) confirmed the presence of a well-defined collagen membrane with viable cells distributed along the inner shell. The nucleus-rich zones (stained dark blue/purple by hematoxylin) clearly delineated the cellular layer adherent to the collagen scaffold, while the eosinophilic (pink) regions indicated the presence of extracellular matrix components. The histological sections showed that cells not only adhered to the pre-existing collagen shell but also actively deposited additional ECM, resulting in a thickened, matrix-enriched tissue layer reminiscent of native connective tissue. This de novo matrix production is a hallmark of functional tissue remodeling and demonstrates that the CMC microenvironment supports cell-mediated ECM synthesis and maturation [34].

The capsule structure remained intact without evidence of shell collapse, fragmentation, or core refilling by gel contraction. The hollow lumen was preserved even after 14 days of culture, confirming the exceptional long-term structural stability of the collagen shell. This stability is critical for maintaining a consistent microenvironment and ensuring reproducibility in long-term culture experiments or potential therapeutic applications.

#### 2.4.3. Biological Implications

The combination of (i) sustained cell viability, (ii) shell-localized 3D cellular organization, and (iii) active extracellular matrix deposition within CMCs demonstrates that this platform successfully recapitulates key features of native tissue architecture. Unlike conventional core-filled hydrogel carriers, where cells are distributed throughout a dense matrix with limited mobility and interaction, the hollow-shell configuration of CMCs provides cells with a spatially organized, biomimetic microenvironment. The adherent cells can proliferate, migrate, and deposit ECM along the inner surface of the collagen shell, forming a tissue-like structure with hierarchical organization. This capacity for tissue-like remodeling is particularly relevant for applications in regenerative medicine, organoid engineering, and in vitro disease modeling, where the goal is to recreate physiologically relevant tissue architecture and cell–matrix interactions.

## 3. Conclusions

This study demonstrates the first successful development of hollow-shell collagen microcapsules (CMCs) using a gelatin sacrificial template approach for three-dimensional cell culture. This novel platform overcomes critical limitations of conventional alginate-based carriers—poor molecular permeability, lack of cell adhesion motifs, and structural instability—through the combination of hollow architecture and collagen’s intrinsic bioactivity. CMCs exhibited 2.5-fold enhancement in molecular diffusion, maintained structural integrity for 14 days compared to the 3-day collapse of alginate controls, and supported superior cell proliferation with tissue-like organization and extracellular matrix deposition. These quantitative improvements represent a significant advance over existing cell encapsulation technologies.

The CMC system provides a biologically favorable microenvironment with enhanced mass transport properties, positioning it as a promising platform with potential applications in tissue engineering, regenerative medicine, and drug screening. Further validation studies including in vivo assessment and clinical translation research will be necessary to fully realize the therapeutic potential of this technology.

## 4. Materials and Methods

### 4.1. Materials

Type I native collagen derived from porcine skin (I-AC, 5 mg/mL) was purchased from Koken Co., Ltd. (Tokyo, Japan) and used without further purification. Gelatin powder (porcine skin-derived, bloom number ~300) was obtained from Nitta Gelatin Inc. (Osaka, Japan). Sodium alginate (low viscosity) was purchased from Nacalai Tesque, Inc. (Kyoto, Japan). FITC-labeled dextran (4 kDa and 70 kDa) was obtained from Sigma-Aldrich (St. Louis, MO, USA). Cell Counting Kit-8 (CCK-8) was purchased from Dojindo Laboratories (Kumamoto, Japan). All other reagents were of analytical grade and used as received.

### 4.2. Cell Culture

Mouse fibroblast NIH 3T3 cells were cultured routinely in standard culture medium (Dulbecco’s Modified Eagle Medium supplemented with 10% fetal bovine serum and 1% penicillin-streptomycin) at 37 °C in a humidified atmosphere containing 5% CO_2_.

### 4.3. Production of Cell-Encapsulating Carriers

#### 4.3.1. Collagen Microcapsules (CMCs)

**Figure 5:**
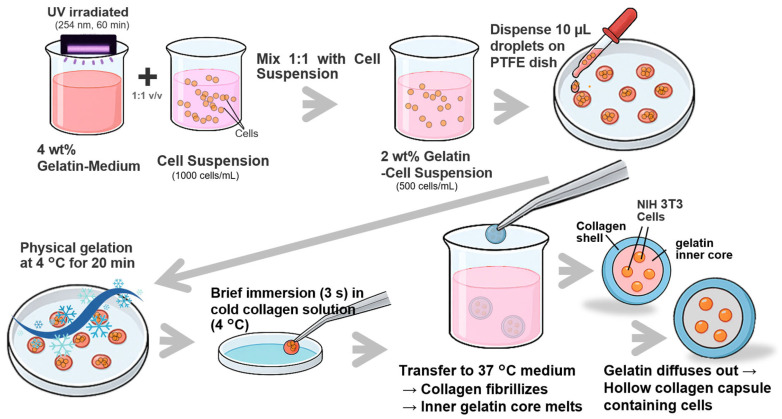
Schematic illustration of the preparation process of collagen microcapsules (CMCs).

To fabricate hollow collagen microcapsules (CMCs) encapsulating NIH 3T3 cells, a thermally induced phase-separation approach utilizing a gelatin sacrificial template was employed (Figure 5). First, a 4 wt% gelatin-medium solution was prepared by dissolving gelatin powder in culture medium, followed by UV sterilization (254 nm, 60 min) and thermal equilibration at 37 °C. NIH 3T3 cells were suspended in culture medium at a density of 1.0 × 10^6^ cells/mL and mixed in a 1:1 volumetric ratio with the 4 wt% gelatin-medium solution to obtain a 2 wt% gelatin-cell suspension containing 5.0 × 10^5^ cells/mL. Ten-microliter droplets of this suspension were manually dispensed onto a PTFE-coated culture dish (hydrophobic surface) and subjected to physical gelation at 4 °C for 20 min to form solid gelatin beads. The gelled beads were then briefly immersed (~3 s) in a cold (4 °C) collagen solution (5 mg/mL in 10 mM HCl, neutralized to pH 7.4 with 10× phosphate-buffered saline (PBS)) to coat the bead surface with a collagen layer. Following this coating step, the beads were immediately transferred to pre-warmed culture medium (37 °C, pH 7.4). At 37 °C, the collagen shell underwent fibrillogenesis and formed a stable, gelled membrane around the gelatin core. Simultaneously, the inner gelatin core, which remains solid below ~25 °C, liquefied and gradually diffused outward through the porous collagen network, leaving behind a hollow lumen enclosed by a cell-laden collagen shell. The resulting CMCs were maintained under standard culture conditions (37 °C, 5% CO_2_) for subsequent experiments.

#### 4.3.2. Production of Alginate–Calcium Capsules (Core-Filled Type, Alg-Beads)

Alginate–calcium hydrogel capsules (Alg-Beads) containing NIH 3T3 cells [35] were prepared to serve as a comparative control. Briefly, a 2 wt% sodium alginate solution was prepared in culture medium, and NIH 3T3 cells were suspended in this alginate solution at a density of 5.0 × 10^5^ cells/mL. Ten-microliter droplets of this cell-laden alginate suspension were manually dispensed into a 100 mM CaCl_2_ solution, where they immediately gelled via ionic crosslinking. The resulting Alg-Beads were incubated in the CaCl_2_ solution for 10 min to ensure complete gelation, then washed three times with PBS, and finally transferred to standard culture medium for parallel experiments with CMCs.

### 4.4. Cell Proliferation Assay

Cell proliferation within CMCs and Alg-Beads was evaluated using the CCK-8 assay (Dojindo Laboratories, Kumamoto, Japan). At predetermined time points (5 h, Day 1, Day 3, Day 5, Day 7, and Day 10 post-encapsulation), capsules (*n* = 6 per time point) were transferred to a 96-well plate containing 100 µL of culture medium. Ten microliters of CCK-8 reagent was added to each well, and the plate was incubated at 37 °C for 2 h. The absorbance at 450 nm was measured using a microplate reader (Bio-Rad, Hercules, CA, USA). Higher absorbance values correspond to greater metabolic activity and cell number.

### 4.5. Microscopy, Morphological Observation, and Capsule Shell Thickness Measurement

The morphology and cellular distribution within CMCs and Alg-Beads were observed using an inverted phase-contrast microscope (Leica DMI3000 B, Leica Microsystems, Wetzlar, Germany) at predetermined time points. For quantitative analysis of membrane thickness, digital images of CMCs (*n* = 100) were acquired at Day 0 immediately after fabrication. The shell thickness was measured at multiple locations around the capsule circumference using ImageJ software (ver.1.54h, National Institutes of Health, Bethesda, MD, USA), and the thickness distribution was analyzed by generating a histogram.

### 4.6. Histological Analysis

For histological examination, CMCs were collected on Day 14. Samples were fixed in 10% (*v*/*v*) neutral-buffered formalin at room temperature for 24 h and washed three times with PBS. Fixed samples were dehydrated through a graded ethanol series (70%, 80%, 90%, 95%, and 99.5%; 30 min each), cleared in xylene (2 × 30 min), and embedded in paraffin (Paraplast Plus, Leica Biosystems, Wetzlar, Germany). Serial sections (5 µm thick) were cut using a sliding microtome (Retoratome REM-710, Yamato Kohki industrial Co., Ltd., Saitama, Japan) and subsequently deparaffinized, rehydrated, and stained with hematoxylin (8 min) and eosin (8 min). Stained specimens were observed under a light microscope (Leica DMI3000 B, Leica Microsystems, Wetzlar, Germany) equipped with a digital camera for image acquisition.

### 4.7. Permeability Measurements Using FITC–Dextran Diffusion

The permeability of the capsule membranes was evaluated by monitoring the inward diffusion of FITC–dextran with two different molecular weights (4 kDa and 70 kDa; Sigma-Aldrich, USA). Cell-free CMCs and Alg-Beads were immersed in phosphate-buffered saline (PBS, pH 7.4) containing 0.5 mg/mL FITC–dextran and incubated at 37 °C in the dark for up to 4 h. At predetermined time points (0, 1, 2, 3, and 4 h), samples were gently washed with PBS to remove unbound probe on the surface and immediately imaged. Fluorescence images were acquired using an inverted fluorescence microscope (Leica DMI3000 B, Leica Microsystems, Germany) equipped with a GFP filter set (excitation 490 nm, emission 525 nm). For CMCs, fluorescence was quantified inside the hollow lumen, whereas for Alg-Beads the fluorescence intensity was measured in the central region of the hydrogel interior. Image analysis was performed using ImageJ, and the mean fluorescence intensity inside the capsules (*I*_in_) and that of the external solution (*I*_out_) were measured to calculate the partition coefficient (Partition coefficient = *I*_in_/*I*_out_). To further determine the effective diffusion coefficient (*D*), time-resolved radial fluorescence intensity profiles were extracted from the capsule periphery toward the center (radius_pixel = 0 at the outer surface). The temporal evolution of fluorescence intensity *I*(*r*, *t*) was fitted using Fick’s second law for diffusion in a semi-infinite medium:

*I*(r,t) = *I*_0_ + *A* · erfc(*r*/2√(*Dt*))

where *r* is the radial distance from the capsule surface, *t* is time, *I*_0_ is the baseline fluorescence, and *A* is a scaling constant for each time point. The capsule radii (in µm) were determined from microscopic images and converted to metric units using calibrated pixel sizes. The fitting was performed using Python (ver.3.14.2, SciPy, NumPy), and the diffusion coefficient *D* was obtained by global least-squares fitting across all time points. Stokes–Einstein diffusion coefficients in water were calculated for comparison using reported hydrodynamic radii (4 kDa: ~4.5 nm; 70 kDa: ~6.0 nm).

### 4.8. Statistical Analysis

All quantitative data are presented as mean ± standard deviation (SD). Normality was assessed using the Shapiro–Wilk test, and variance homogeneity was verified by Levene’s test. Comparisons between two groups were performed using Student’s *t*-test. Statistical significance was set at *p* < 0.01.

## Figures and Tables

**Table 1:** Diffusion coefficients of FITC–dextran (4 kDa and 70 kDa) in CMCs and Alg-Beads.

Sample	Molecular Weight	D (µm^2^/s)		R^2^
CMCs	4 kDa	219.2	±2.9	0.96
Alginate	4 kDa	98.8	±2.3	0.91
CMCs	70 kDa	66.4	±0.6	0.98
Alginate	70 kDa	69.8	±2.2	0.94

## Data Availability

The data presented in this study are available on request from the corresponding author.

## Supplementary Materials

The following supporting information can be downloaded at: https://www.mdpi.com/article/10.3390/gels12010015/s1, Figure S1: Representative optical microscopy images of collagen microcapsules (CMCs) demonstrating the hollow-shell architecture and reproducibility of the fabrication process.
