# Supplementary material for: Development and Characterization of Hollow-Shell Collagen Microcapsules for Three-Dimensional Cell Culture
Source: Gels. 2025 Dec 24;12(1):15. doi: 10.3390/gels12010015 (PMC12840939; doi:10.3390/gels12010015)
Supplement: Supplementary file 1 [file gels-12-00015-s001.zip › gels-4018189-supplementary.pdf]

Supplementary Materials

Development and Characterization of Hollow-Shell Collagen Microcapsules for Three-Dimensional Cell Culture

Yusuke Chiwata [1], Shigehisa Aoki [2], Takehisa Sakumoto [2] and Takayuki Narita [1,*]

[1] Department of Chemistry and Applied Chemistry, Faculty of Science and Engineering, Saga University, Saga 840-8502, Japan
[2] Department of Pathology and Microbiology, Faculty of Medicine, Saga University, Saga 840-8502, Japan

* Correspondence: naritat@cc.saga-u.ac.jp

Supplementary Figure S1

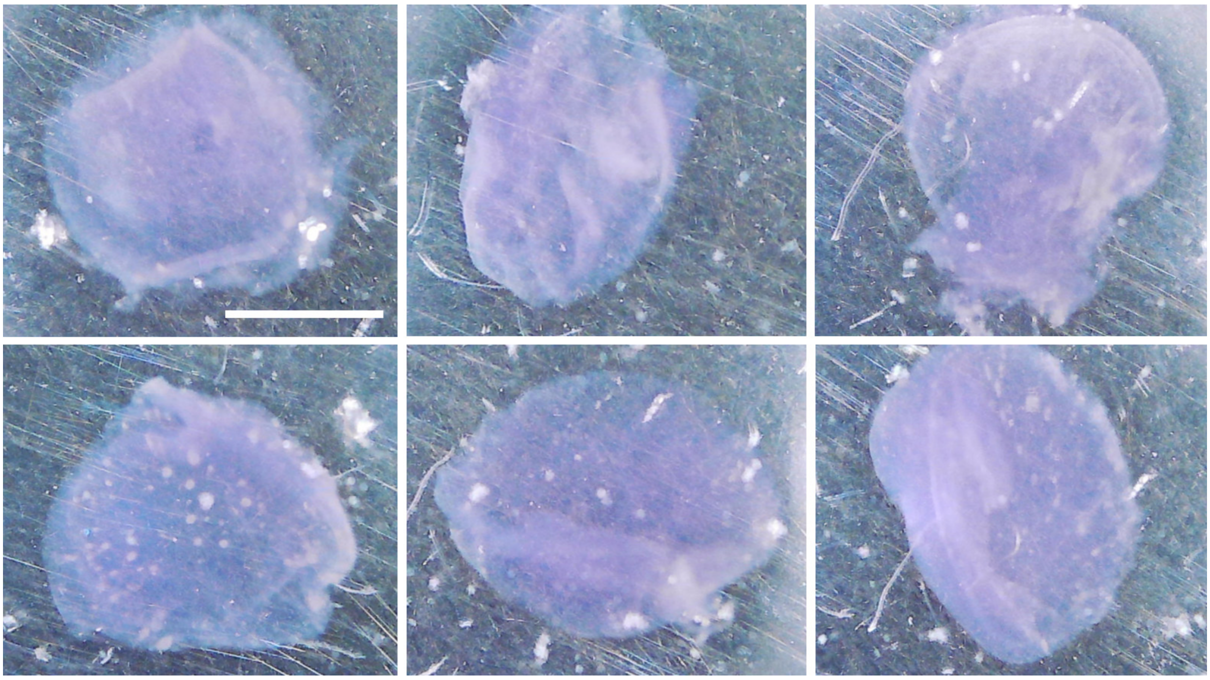

Figure S1. Representative optical microscopy images of collagen microcapsules (CMCs) demonstrating the hollow-shell architecture and reproducibility of the fabrication process. Six individual CMCs are shown, each exhibiting the characteristic spherical morphology and transparent appearance indicative of the hollow lumen structure achieved using the gelatin sacrificial template method. The images illustrate the excellent reproducibility in capsule size and shape, with all capsules maintaining their structural integrity. These hollow-shell structures are ready for cell encapsulation and 3D culture applications as demonstrated in the main manuscript (Figures 1A, 4, and 5). The capsules shown here are prior to cell encapsulation. Scale bar: 1 mm (shown in upper left panel).
